# miR-378 suppresses the proliferation, migration and invasion of colon cancer cells by inhibiting SDAD1

**DOI:** 10.1186/s11658-017-0041-5

**Published:** 2017-07-17

**Authors:** Mingxi Zeng, Linlin Zhu, Liangping Li, Changming Kang

**Affiliations:** 1 Department of Digestive Diseases, Sichuan Provincial People’s Hospital, No. 32 Yihuan Road, Qingyang District, Chengdu, Sichuan Province 610072 China; 2 Department of Digestive Diseases, West China Hospital of Sichuan University, Chengdu, 610041 China

**Keywords:** miR-378, Colon cancer, Proliferation, Migration, Invasion, SDAD1, Wnt/β-catenin

## Abstract

**Background:**

MicroRNAs (miRNAs) play important roles in the growth and metastasis of colon cancer. It is known that one set of miRNAs are dysregulated in colon cancer cells, but the mechanism of their role in cancer development is still largely unknown. Our study focuses on the role of miR-378 in colon cancer cells.

**Methods:**

Human colon cancer tissues and adjacent non-tumor tissues were collected from patients diagnosed in pathological examinations. In addition, human colon cancer cell lines LoVo, CaCo2, SW1116, SW480 and HCT-116, and a normal colonic mucosa cell line NCM460 were included. Quantitative RT-PCR was used to detect the miR-378 level in the clinical tissues and cell lines. In SW480 and HCT-116, miR-378 was artificially overexpressed or suppressed. Cell viability and proliferation were measured using MTT and colony formation assays, and apoptosis was detected via annexin V-PI staining and flow cytometry analysis. The transwell technique was applied to detect the migration and invasion of the colon cancer cells, and their epithelial–mesenchymal transition (EMT) was evaluated by detecting EMT-associated markers using Western blotting. Bioinformatics methods were used to predict the potential targets of miR-378, and luciferase reporter assays were performed to conform the direct binding between miR-378 and its target mRNA. The activity of the Wnt/β-catenin pathway was evaluated by detecting the key factors through Western blotting.

**Results:**

We found that miR-378 expression was low in colon cancer tissues and cell lines. Overexpression of miR-378 not only inhibits the proliferation of colon cancer cells in vitro by inducing apoptosis, but also inhibits migration and invasion by inhibiting the EMT of colon cancer cells. SDAD1 is a direct target gene of miR-378, and knockdown of SDAD1 suppresses the proliferation, migration and invasion of colon cancer cells. We also confirmed that miR-378 alleviated the malignant phenotypes of colon cancer cells by inhibiting the Wnt/β-catenin pathway.

**Conclusion:**

miR-378 inhibits the proliferation, migration and invasion of colon cancer cells by targeting SDAD1, defining miR-378 as a potential target for the diagnosis and treatment of colon cancer.

## Background

In the worldwide rankings of cancer incidence and mortality, colon cancer ranks third and fourth, respectively [1]. There are more than one million new colon cancer cases and about 700,000 people die of colon cancer each year [2]. Multiple factors are involved in the occurrence and development of colon cancer, including the activation of oncogenes and inactivation of tumor suppressor genes [3]. The mutation in the tumor suppressor gene is crucial for the transition from non-invasive to invasive disease. Mutations are found in invasive colorectal cancer (CRC) with an increasing frequency (75%) correlating to the extent of malignance [4]. Point mutations in the K-ras gene can lead to amino acid substitutions in the protein, activating the potential carcinogenicity of the *ras* gene [5].

Many research groups explored the biological mechanisms of colon cancer, identifying many oncogenes and tumor suppressor genes. Emerging evidence suggests that some changes in the expressions of genes regulating oncogenes or tumor suppressor genes can also lead to the occurrence of colon cancer. A clearer understanding of the mechanism of colon cancer occurrence, development, migration and recurrence coupled with exploration of the new molecular markers of colon cancer would aid greatly in the early diagnosis and treatment of colon cancer.

MicroRNAs (miRNAs) are endogenous non-coding small RNAs that are about 19–24 nt in length and play important roles in plants and animals by targeting the 3-terminal non-coding region of the target gene mRNA to cause transcriptional repression or regulate mRNA degradation [6–8]. An increasing number of studies have shown that miRNAs play important roles in promoting or inhibiting tumor cell proliferation, invasion, apoptosis and drug resistance by regulating oncogenes or tumor suppressor genes [9–11].

It has been found that miRNAs are differentially expressed in colon cancer cells. They are closely related to the biological and clinical characteristics of colon cancer and play an important role in its development and progression [12]. For example, miR-582-5p inhibits the expression of APC in colon cancer cells by targeting the 3′ untranslated region (3’UTR) region of the gene, thereby promoting proliferation [13]. miR-203 suppresses proliferation by decreasing the expression level of EIF5A2 [14]. Several studies indicate that miRNAs are involved in the metastasis of colon cancer. miR-552 promotes the migration of colon cancer cells by targeting ADAM28 [15] and miR-9 inhibits colon cancer cell migration and invasion by downregulating TM4SF1 [16]. Based on such results, miRNAs are currently in focus as a potential target for colon cancer therapy.

miR-378 has been reported to play an important role in many types of cancer. For example, it can reverse drug resistance to cisplatin in lung cancer [17]. In gliomas, decreased miR-378 levels indicate high tumor invasiveness and poor prognosis [18]. miR-378 inhibits the growth and proliferation of tumor cells in hepatocellular carcinoma [19]. However, there are few studies on the relationship between miR-378 and the development of colon cancer.

In this study, we found that miR-378 plays a tumor suppressor role in colon cancer cells. We also confirmed that SDAD1 is a direct target gene for miR-378, and that SDAD1 promotes the proliferation, migration and invasion of colon cancer cells. In addition, we found that miR-378 suppresses the malignant behavior of colon cancer cells by inhibiting Wnt/β-catenin pathway. These data suggest that miR-378 may be a target for colon cancer diagnostics and treatment.

## Methods

### Colon cancer tissue samples

Twenty-seven pairs of colon cancer tissues and adjacent non-tumor tissues were collected from patients diagnosed in our hospital from August 2014 to September 2015. All patients who provided samples had been diagnosed in pathological examinations and not treated with radiotherapy and chemotherapy before surgery. Informed consent was obtained from each patient, and all the experiments were approved by the Ethics Committee of Sichuan Provincial People’s Hospital. All tissue samples were maintained in liquid nitrogen.

### Cell lines and cell culture

The human colonic carcinoma cell lines LoVo, CaCo2, SW1116, SW480 and HCT-116, and the normal colonic mucosa cell line NCM460 were purchased from the Shanghai Cell Bank. All cell lines were cultured in DMEM medium containing 10% FBS, 100 U/ml penicillin and 100 mg/ml streptomycin at 37 °C in a 5% CO_2_ incubator.

### RNA extraction and quantitative RT-PCR analysis

Total RNA was extracted from colonic cancer cells and tissue samples according to the instructions for the mirVANA RNA isolation Kit (Ambion). The extraction concentration was measured with a NanoDrop spectrophotometer. Standby preservation at −80 °C. For quantitative RT-PCR, RNA was reverse transcribed into cDNA using M-MLV reverse transcriptase and RiboLock nucleic acid enzyme inhibitor (Applied Biosystems). The GenePharma SYBR Green method was used to conduct real-time PCR with Bio-Rad IQ-5 with the reaction consisting of a 10-min hot start at 95 °C, then 50 cycles of 15 s at 94 °C, 30 s at 55 °C, and 30 s at 70 °C. The 2^-ΔΔCt^ method was used for mRNA or miRNA quantification analysis, with β-actin or U6 as standards, respectively. The primer sequences are shown in Table 1.

**Table 1:** Sequence of primers used in this study

Name	Sequence (5′ to 3′)
miR-378-RT	GTCGTATCCAGTGCAGGGTCCGAGGTGCACTGGATACGACGCCTTC
U6-RT	GTCGTATCCAGTGCAGGGTCCGAGGTGCACTGGATACGACAAAATATGG
miR-378-Fwd	TGCGGACTGGACTTGGAGTCAG
U6-Fwd	TGCGGGTGCTCGCTTCGGCAGC
miRNA-Rvs	CCAGTGCAGGGTCCGAGGT
SDAD1-Fwd	AAAACAGTTGGCACTACGAG
SDAD1-Rvs	CCTACCATTAGCCGTCTCC
β-actin-Fwd	TTGCGTTACACCCTTTCTTG
β-actin-Rvs	TGCTGTCACCTTCACCGTTC

### Plasmid, ASO-miRNA, and siRNA transfection

Colon cancer cells were seeded in 6-well plates overnight (1 × 10^6^ per well). Cells were transfected with miR-378 mimics or ASO-miR-378 (GenePharma) using Lipofectamine 2000 (Invitrogen) according to the manufacturers’ instructions, then transfected with SDAD1 siRNA (si-SDAD1, GenePharma). At 6 h after transfection, the cells were cultured in normal culture medium. Analyses were performed 48 h later.

### Western blotting

The cells showing good growth were lysed by RIPA. Then 50 μg samples were loaded in 10% SDS-PAGE electrophoresis. Next, the protein on the separation gel was transferred onto PVDF film via electrotransformation and blocked with 5% blotto. Two hours after being blocked, the protein was incubated with primary antibody (rabbit anti-human polyclonal antibody, 1:500) and cultured overnight at 4 °C. When it was equilibrated to room temperature, the film was washed, and the secondary antibody (goat anti rabbit, 1:1000) was added. The membrane was put into Lightning Chemiluminescence Reagent for 2 min, then removed and put into the exposure box. Photographic film was exposed in the darkroom for a minute, followed by development and fixing. LabWorks gel-imaging and analysis system was adopted for photographing and analyzing the luminance value of each group of target band.

### Luciferase reporter assay

An amount of 1 × 10^4^ cells were seeded in 48-well plates. After 24 h, SW480 cells were co-transfected with 200 ng miR-378 and 50 ng SDAD1–3’UTR plasmids. Cells were lysed 36 h after transfection. Fluorescence activity was measured with a dual luciferase system (Promega).

### Cell proliferation analysis

MTT assay was used to analyze the proliferation of colon cancer cells. Colon cancer cell lines were seeded in 96-well plates at a rate of 3000 cells/well for 24, 48 and 72 h, respectively, and 10 μl MTT (Sigma) was added to each well. After 4–6 h incubation, the medium was discarded. Then 100 μl DMSO was added to each well and the cells were incubated in the dark for 20 min. The absorbance at 570 nm was measured using an enzyme-labelling measuring instrument. Each group consisted of 5 duplicates, and at least three separate experiments were repeated.

### Colony formation analysis

Transfected colon cancer cells were treated with trypsin–collagenase to digest them into a single cell suspension, then seeded into 12-well plates at a rate of 400 cells/well and cultured in a 5% CO_2_ incubator at 37 °C for 14 days. The cells were washed twice with PBS and then stained with 2% crystal violet for 15 min, and the plate was dried at room temperature. The number of clones formed in the plate was counted. At least three separate experiments were carried out.

### Apoptosis analysis

Apoptosis was analyzed via flow cytometry using annexin V-PI staining. Briefly, 1 × 10^5^ cells were seeded in 6-well plates, treated with PBS twice 48 h after transfection, digested and resuspended with 100 μl binding buffer. The cell density was adjusted to 0.5 × 10^6^/100 μl cells. 5 μl Annexin V/FITC was added to stain the cells for 10 min in the dark at room temperature. Then 100 μl binding buffer was added and mixed by flicking. Next, the cells were stained with 5 μl PI for 5 min in the dark at room temperature. Apoptosis rates were analyzed using a FACScan flow cytometry (BD Biosciences) within 1 h [20].

### Transwell assays

The aperture in the bottom membrane of Transwell chambers (Corning) was 8 μm. The chambers were coated with matrigel (Sigma) for the determination of invasive ability. No matrigel was added for the migration test. The under layer was filled with 600 μl DMEM nutrient solution containing 10% FBS. The volume of the upper layer of the Transwell chambers was 200 μl. This was inoculated with 5 × 10^5^ colonic cancer cells. The cells were cultured in an incubator at 37 °C with 5% CO_2_ for 48 h, then the wells were removed and fixed in liquid consisting of methanol and glacial acetic acid (3:1), for 30 min. Then the wells were washed with PBS, stained with 0.1% crystal violet and finally mounted. For the observation, 5 random fields were randomly selected and the numbers of stained cells were counted under a microscope [21].

### Statistical methods

GraphPad Prism 5.0 (GraphPad Software) statistical software was used. Data are presented as means ± standard deviation. Statistical analyses were performed with Student’s *t*-test with *p* < 0.05 considered statistically significant.

## Results

### miR-378 is expressed at a low level in colon cancer

**Fig. 1:**
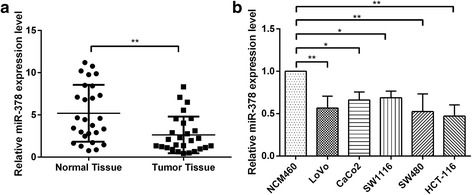
miR-378 is downregulated in CRC samples and cell lines. **a** Expression of miR-378 in 27 CRC samples was compared with that in adjacent non-tumor tissues; determination via quantitative RT-PCR. U6 snRNA served as an internal control. **b** Quantitative RT-PCR was performed to detect the expression of miR-378 in 5 CRC cell lines (LoVo, CaCo2, SW1116, SW480, HCT-116) and a normal colonic cell line (NCM460). *p < 0.05, **p < 0.01

To determine the miR-378 level in colon cancer, we first determined the expression levels of miR-378 in 27 pairs of colon cancer tissues and adjacent normal tissues using quantitative RT-PCR. As shown in Fig. 1a, miR-378 has a low level of expression in colon cancer tissues. Next, we detected the expression level of miR-378 in colon cancer cell lines. Compared to the expression level in the normal colonic epithelial cell line NCM460, miR-378 has a low level of expression in multiple colon cancer cell lines, including LoVo, CaCo2, SW1116, SW480 and HCT-116 (Fig. 1b).

### miR-378 inhibits proliferation of colon cancer cells by inducing apoptosis

**Fig. 2:**
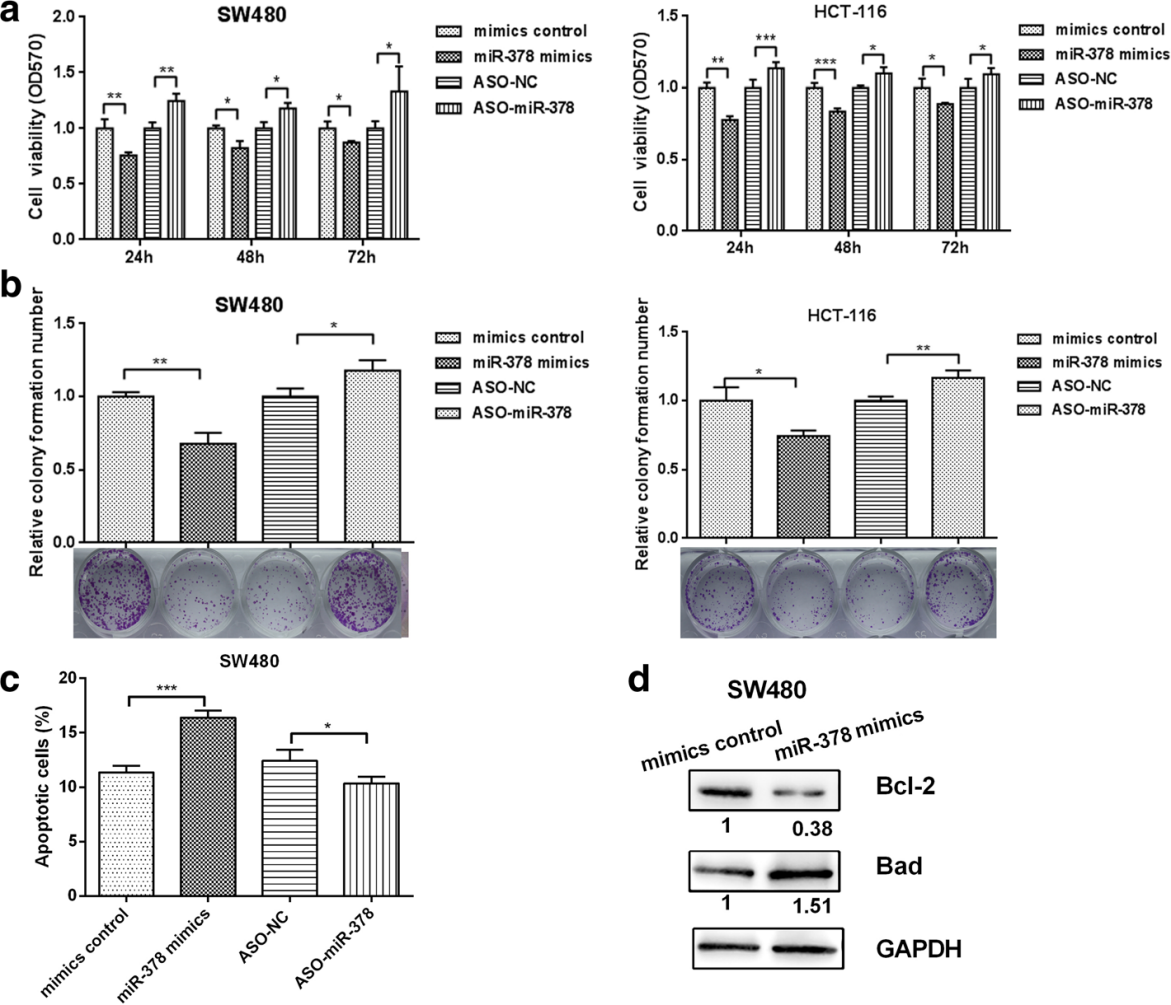
Influences of miR-378 on the proliferation of colon cancer cells. **a** The MTT assay was performed to measure the amount of SW480 and HCT-116 cells transfected with miR-378 mimics or ASO-miR-378. **b** A colony formation assay was performed to detect SW480 and HCT-116 cells transfected with miR-378 mimics or ASO-miR-378. **c** FACS was performed to determine the level of cell apoptosis when miR-378 was overexpressed or knocked down. **d** Western blot was used to detect the apoptosis proteins Bad and Bcl-2 in SW480 cells transfected with miR-378 mimics. GAPDH was used as a loading/transfer control and for normalization of the values. *p < 0.05, **p < 0.01, ***p < 0.001

To further explore the role of miR-378 in colon cancer cells, SW480 and HCT-116, which had low expression levels of miR-378, underwent further study. First, we used MTT to detect the proliferation of colon cancer cells. As shown in Fig. 2a, overexpression of miR-378 significantly inhibited the proliferation of colon cancer cells, and blocking miR-378 could promote the proliferation of colon cancer cells. Next, the colony formation assay also showed that overexpression of miR-378 inhibited the proliferation capacity by about 25% (Fig. 2b).

We then used flow cytometry to detect colon cancer cell apoptosis. As shown in Fig. 2c, overexpression of miR-378 significantly increased apoptosis in colon cancer cell line SW480, whereas the level of apoptosis decreased when miR-378 was blocked. Western blot was used to detect the expression of the proapoptotic gene Bad and anti-apoptotic/apoptosis-suppressing gene Bcl-2. As shown in Fig. 2d, the expression level of Bad increased by 51% while the expression level of Bcl-2 decreased by 48% when miR-378 was overexpressed in SW480 cells. We believe that miR-378 inhibits the proliferation of colon cancer cells by inducing apoptosis.

### miR-378 inhibits colon cancer cell migration and invasion

**Fig. 3:**
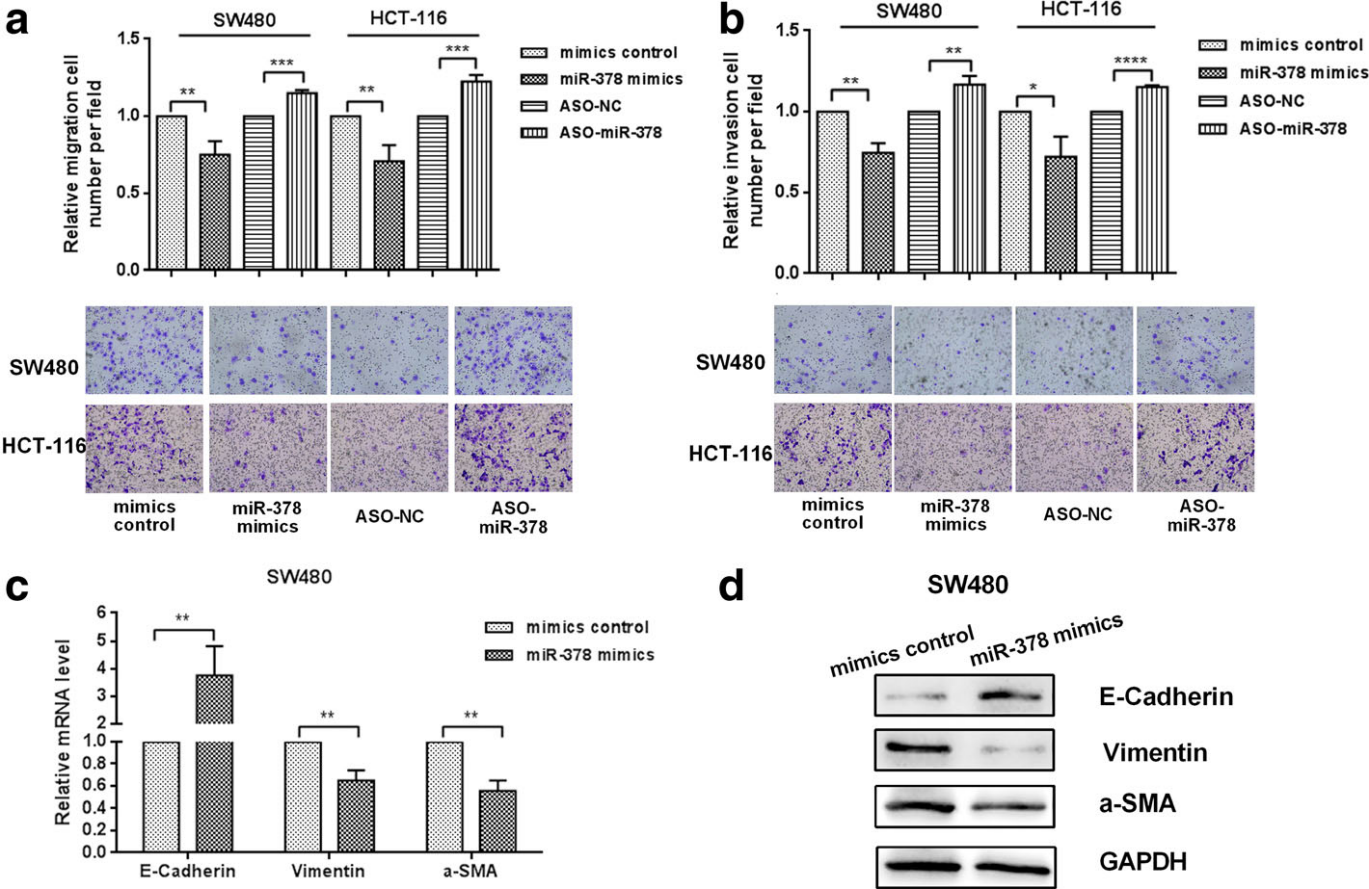
The effect of miR-378 on the migration and invasion abilities of colon cancer cells. **a**, **b** The effect of miR-378 mimics on the migration (**a**) and invasion (**b**) of SW480 and HCT-116 cells determined using Transwell plates. **c** Quantitative RT-PCR was used to measure the mRNA levels of three EMT markers, e-Cadherin, vimentin and a-SMA, in miR-378 mimics transfected SW480 cells. **d** Western blot was used to measure the protein levels of these EMT makers in SW480 cells transfected with miR-378 mimics. *p < 0.05, **p < 0.01, ***p < 0.001, ****p < 0.0001

We used Transwell migration and invasion assays to investigate the effect of miR-378 on the migration and invasion of colon cancer cells. As shown in Fig. 3a, overexpression of miR-378 in colon cancer cell lines SW480 and HCT-116 inhibited cell migration significantly (by approximately 25%). Overexpression of miR-378 inhibited cell invasion (Fig. 3b). Conversely, knockdown of miR-378 promoted migration and invasion of colon cancer cells. To study the underlying mechanism, we used quantitative RT-PCR and Western blot to detect the mRNA and protein expression levels of the marker proteins related to the process of epithelial–mesenchymal transition (EMT). The results indicated that overexpression of miR-378 could inhibit the EMT process of colon cancer cells (Fig. 3c and d).

### SDAD1 is a direct target gene for miR-378

**Fig. 4:**
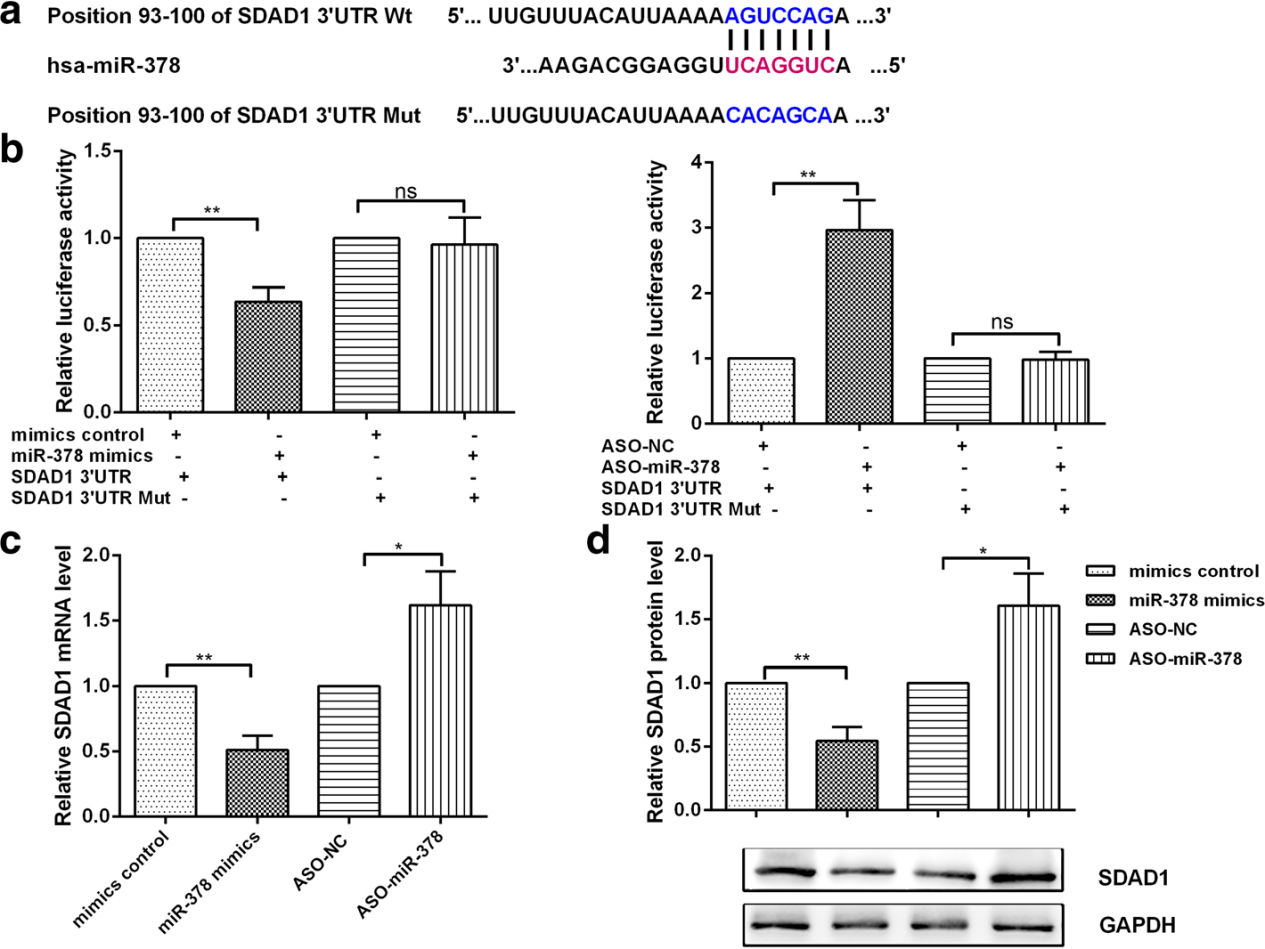
SDAD1 is a direct target of miR-378. **a** The complementary sequences of the miR-378 binding site in SDAD1 3’UTR. The sequence of mutated SDAD1 3’UTR is also shown. **b** The effect of miR-378 on luciferase intensity controlled by the wild type (Wt) or mutant (Mut) 3’UTR of SDAD1 was determined using the luciferase assay. **c**, **d** The mRNA (C) and protein (D) levels of SDAD1 were detected using quantitative RT-PCR and western blot, respectively, in SW480 cells transfected with miR-378 mimics. *p < 0.05, **p < 0.01, ns: not significant

We searched for miR-378 target genes using the TargetScan database and related functional analyses. SDAD1 was chosen as the object for further study (Fig. 4a). To demonstrate that SDAD1 is a direct target gene for miR-378, we constructed the fluorescent reporter vectors with the miR-378 binding site on SDAD1–3’UTR. By co-transfecting miR-378 with the SDAD1–3’UTR reporter vector into colon cancer cells, we detected the activity of luciferase to demonstrate the condition of miR-378 targeting SDAD1. At the same time, we made point mutations in SDAD1–3’UTR to construct mutant SDAD1–3’UTR fluorescence reporter plasmid. Overexpression of miR-378 resulted in decreased fluorescence activity and no significant changes in fluorescence activity were observed in mutant UTR (Fig. 4b). This demonstrates that miR-378 and SDAD1 have a direct and negative regulatory relationship. We also found the expression levels of mRNA and protein in SDAD1 respectively decreased by 49% and 50% when miR-378 was overexpressed, while the mRNA and protein levels of SDAD1 respectively increased 4 and 1.5 times when blocking miR-378 (Fig. 4c and d). These results indicate that miR-378 suppresses expression of SDAD1 in colon cancer cells.

### SDAD1 promotes proliferation and migration/invasion of colon cancer cells

**Fig. 5:**
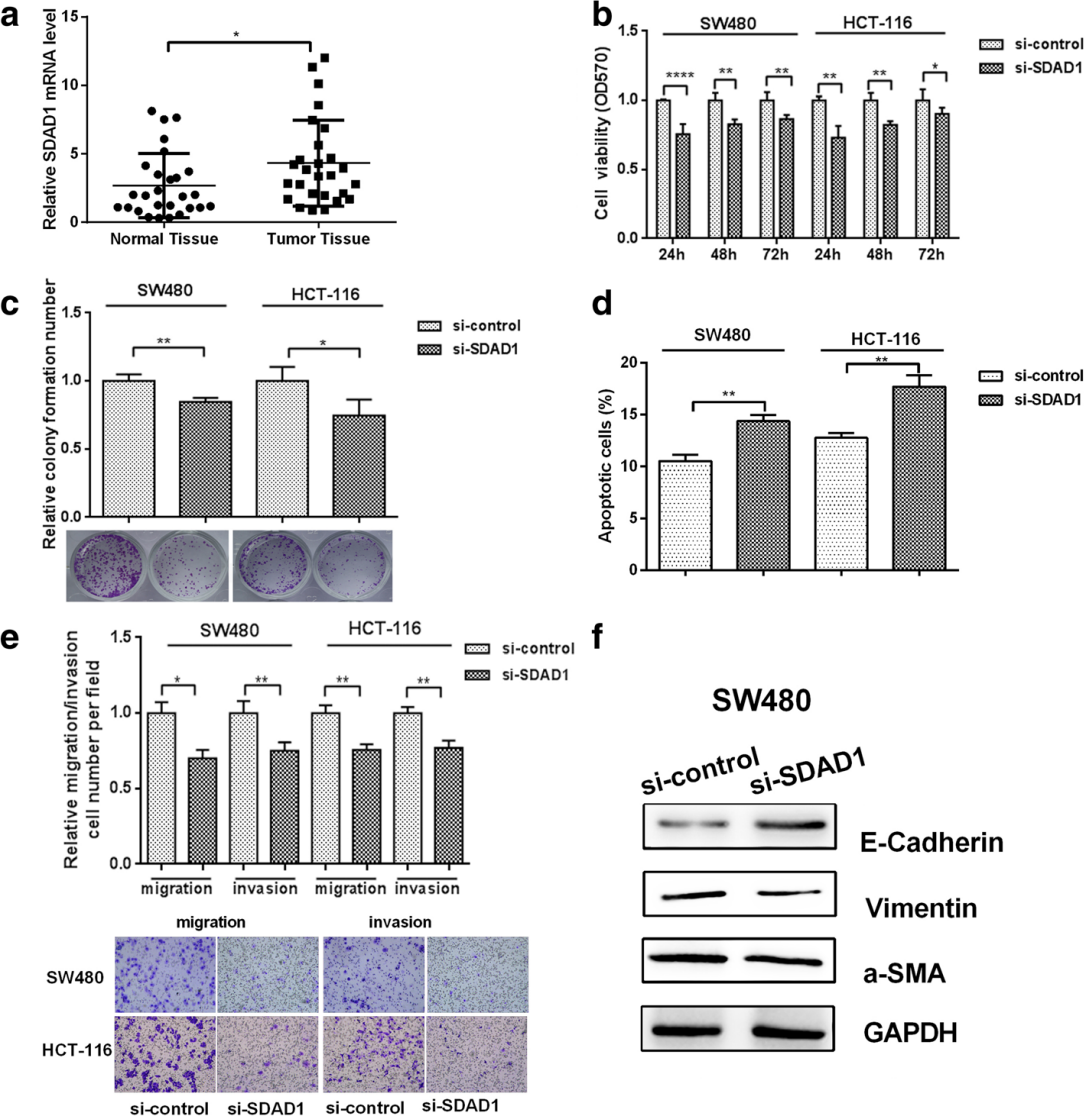
SDAD1 promotes the proliferation, migration and invasion of colon cancer cells. **a** The expression of SDAD1 in 27 pairs of colon cancer and adjacent non-tumor tissues was determined using quantitative RT-PCR. **b** The MTT assay was performed on SW480 and HCT-116 cells transfected with SDAD1 overexpression plasmid. **c** A colony formation assay was performed on SDAD1 overexpression plasmid-transfected SW480 and HCT-116 cells. **d** FACS was performed to analyze the apoptosis of SW480 cells when SDAD1 was overexpressed. **e** The effect of SDAD1 transfection on the migration/invasion of SW480 and HCT-116 cells was determined using Transwell plates. **f** Western blot was used to measure the protein levels of the EMT protein makers in SW480 cells transfected with SDAD1 overexpression plasmid. *p < 0.05, **p < 0.01, ****p < 0.0001

We next explored whether the effects of SDAD1 on the proliferation and migration of colon cancer cells are consistent with this targeting relationship. The SDAD1 level was higher in the colon cancer tissues than in adjacent normal tissues (Fig. 5a). Knockdown of SDAD1 significantly reduced the proliferation of colon cancer cells, suggesting that SDAD1 promotes proliferation of colon cancer cells (Fig. 5b and c). Flow cytometry showed that knockdown of SDAD1 resulted in an increase in apoptosis of SW480 cells by 48% (Fig. 5d). We also investigated the effect of SDAD1 on the migration and invasion of colon cancer cells using Transwell migration and invasion assays. The results showed a decreased migration and invasion of colon cancer cells when SDAD1 was knocked down (Fig. 5e). Also, knockdown of SDAD1 in SW480 colon cancer cells can significantly inhibit the cell EMT process (Fig. 5f). These results indicate that SDAD1 promotes proliferation, migration and invasion of colon cancer cells.

### miR-378 induced Wnt/β-catenin pathway inhibition

**Fig. 6:**
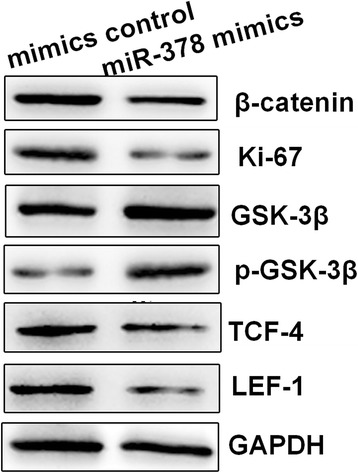
The effect of miR-378 on the Wnt/β-catenin pathway. Western blot was used to measure the levels of β-catenin, Ki-67, GSK-3β, p-GSK-3β, and the transcription factors TCF-4 and LEF-1 when miR-378 was overexpressed

Previous studies have indicated that the Wnt/β-catenin pathway promotes development and progression of colon cancer. Therefore, we continued to investigate whether miR-378 in colon cancer cells inhibits Wnt pathway activation. We determined the expression levels of β-catenin, GSK-3β, p-GSK-3β and Ki-67 in the Wnt/β-catenin pathway in SW480 cells when miR-378 was expressed differently. Overexpression of miR-378 significantly inhibited the expression of β-catenin and Ki-67 (Fig. 6). We also found that overexpression of miR-378 significantly inhibited the expression of two important transcription factors, TCF-4 and LEF-1 (Fig. 6). We believe that miR-378 caused Wnt/β-catenin pathway inhibition.

## Discussion

A tumor is essentially a polygenic disorder where cells escape the normal growth control mechanism, undergoing autonomic proliferation, becoming invasive, and showing a malignant phenotype due to the activation of one or more proto-oncogenes, or the mutation or deletion of a tumor suppressor gene [10].

There are many regulatory factors in the development of cancer. These modulating factors can regulate the expression of oncogenes or tumor suppressor genes to play roles in tumor promotion or inhibition. miRNA has been found to regulate cell proliferation, differentiation and apoptosis by regulating the expression of signaling molecules, such as cytokines, transcription factors, growth factors, pro-apoptotic and anti-apoptotic genes, indicating that miRNA is closely related to tumor occurrence, progression and prognosis.

The dysregulation of miRNA expression may be an important factor in tumor development and progression [22, 23]. Studies have demonstrated that miR-378 has biological functions that can regulate a variety of tumor cells, including cell proliferation [24], migration and invasion [25], and drug resistance [26].

In this study, we found that the expression of miR-378 was significantly decreased in colon cancer specimens and cell lines. We also confirmed that miR-378 can inhibit the proliferation of colon cancer cells. It was also found that miR-378 inhibited the proliferation of colon cancer cells by inducing apoptosis. These results indicate that miR-378 can inhibit the proliferation of colon cancer cells. Furthermore, we found that miR-378 inhibits the migration and invasion of colon cancer cells by inhibiting the epithelial–mesenchymal transition (EMT) pathway, and we further investigated the mechanism of miR-378’s tumor suppressor role in colon cancer cells.

It is well known that miRNAs target the 3′ untranslated region (3’UTR) of the target gene so as to realize its biological function by inhibiting the expression of the target gene [27]. Finding specific miRNAs and their target genes involved in tumorigenesis will provide an important clue for the diagnosis and treatment of malignant tumors in patients.

In our study, we used bioinformatics algorithms to predict that SDAD1 is a candidate target for miR-378. SDAD is a gene family which has not been extensively studied, and the role and mechanism of SDAD1 in cancer have not been reported yet. This is the first identification of an oncogene role for SDAD1 in colon cancer cells. SDAD1 promotes the proliferation of colon cancer cells by reducing apoptosis. Transwell migration and invasion experiments indicated that SDAD1 promotes colon cancer cell migration and invasion abilities by facilitating EMT.

Wnt glycoprotein regulates homeostasis and development by binding to the Frizzled-LRP5/6 complex. The classical function of the Wnt signaling pathway is to induce the stabilization of cytoplasmic β-catenin, nuclear translocation and gene regulation as a T-cell factor (TCF) coactivator. Through the interaction of different signal molecules, Wnt triggers complex signal cascades to promote cell growth, migration, differentiation, development, and so on. The highly conserved Wnt signaling protein family members play an important role in embryonic development. Their abnormal activation can lead to the occurrence of tumors [28]. The abnormality of the Wnt–β-catenin signal pathway is closely related to the occurrence and development of many kinds of tumors. In nasopharyngeal carcinoma and esophageal squamous cell carcinoma, abnormal activation of the Wnt pathway promotes tumor formation [29]. Hepatitis C virus core protein stimulates hepatocyte proliferation due to upregulation of Wnt-1 expression [30].

In our study, we found that miR-378 inhibited the Wnt/β-catenin pathway in colon cancer, which may reduce the malignant progression of colon cancer.

## Conclusion

This study provides new evidence that miR-378 can inhibit the proliferation of colon cancer cells by promoting apoptosis. miR-378 also inhibits the invasion and migration of colon cancer cells by reducing EMT process. miR-378 targets the 3’UTR of SDAD1 to negatively regulate its expression. SDAD1 not only promotes proliferation, migration and invasion but also inhibit apoptosis and promotes EMT of colon cancer cells. This suggests that miR-378 may be a target for the treatment of colon cancer.

## Abbreviations

3’UTR: 3′ untranslated region
cDNA: Complementary DNA
CRC: Colorectal cancer
EMT: Epithelial-mesenchymal transition
miR-378: microRNA-378
miRNA: microRNA
MTT: 3-(4,5-cimethylthiazol-2-yl)-2,5-diphenyl tetrazolium bromide

## References

[1] Meyerhardt JA, Mayer RJ (2005). Systemic therapy for colorectal cancer. N Engl J Med.

[2] Haggar FA, Boushey RP (2009). Colorectal cancer epidemiology: incidence, mortality, survival, and risk factors. Clin Colon Rectal Surg.

[3] Fearon ER, Vogelstein B (1990). A genetic model for colorectal tumorigenesis. Cell.

[4] Bahnassy AA, Zekri AR, Salem SE, Abou-Bakr AA, Sakr MA, Abdel-Samiaa AG (2014). Differential expression of p53 family proteins in colorectal adenomas and carcinomas: prognostic and predictive values. Histol Histopathol.

[5] Bettington M, Walker N, Clouston A, Brown I, Leggett B, Whitehall V (2013). The serrated pathway to colorectal carcinoma: current concepts and challenges. Histopathology.

[6] Valinezhad Orang A, Safaralizadeh R, Kazemzadeh-Bavili M (2014). Mechanisms of miRNA-mediated gene regulation from common downregulation to mRNA-specific upregulation. Int J Genomics.

[7] Bushati N, Cohen SM (2007). microRNA functions. Annu Rev Cell Dev Biol.

[8] Ambros V, Lee RC (2004). Identification of microRNAs and other tiny noncoding RNAs by cDNA cloning. Methods Mol Biol.

[9] Hwang HW, Mendell JT (2006). MicroRNAs in cell proliferation, cell death, and tumorigenesis. Br J Cancer.

[10] Calin GA, Croce CM (2006). MicroRNA signatures in human cancers. Nat Rev Cancer.

[11] Volinia S, Calin GA, Liu CG, Ambs S, Cimmino A, Petrocca F (2006). A microRNA expression signature of human solid tumors defines cancer gene targets. Proc Natl Acad Sci U S A.

[12] Amirkhah R, Schmitz U, Linnebacher M, Wolkenhauer O, Farazmand A (2015). MicroRNA-mRNA interactions in colorectal cancer and their role in tumor progression. Genes Chromosomes Cancer.

[13] Shu Z, Chen L, Ding D (2016). miR-582-5P induces colorectal cancer cell proliferation by targeting adenomatous polyposis coli. World J Surg Oncol.

[14] Deng B, Wang B, Fang J, Zhu X, Cao Z, Lin Q (2016). MiRNA-203 suppresses cell proliferation, migration and invasion in colorectal cancer via targeting of EIF5A2. Sci Rep.

[15] Wang J, Li H, Wang Y, Wang L, Yan X, Zhang D (2016). MicroRNA-552 enhances metastatic capacity of colorectal cancer cells by targeting a disintegrin and metalloprotease 28. Oncotarget.

[16] Park YR, Lee ST, Kim SL, Liu YC, Lee MR, Shin JH (2016). MicroRNA-9 suppresses cell migration and invasion through downregulation of TM4SF1 in colorectal cancer. Int J Oncol.

[17] Chen X, Jiang Y, Huang Z, Li D, Chen X, Cao M (2016). miRNA-378 reverses chemoresistance to cisplatin in lung adenocarcinoma cells by targeting secreted clusterin. Sci Rep.

[18] Li B, Wang Y, Li S, He H, Sun F, Wang C (2015). Decreased expression of miR-378 correlates with tumor invasiveness and poor prognosis of patients with glioma. Int J Clin Exp Pathol.

[19] Niu JX, Meng XK, Ren JJ (2015). Studied microRNA gene expression in human hepatocellular carcinoma by microRNA microarray techniques. World J Gastroenterol.

[20] Ji FJ, Tian XF, Liu XW, Fu LB, Wu YY, Fang XD (2015). Dihydromyricetin induces cell apoptosis via a p53-related pathway in AGS human gastric cancer cells. Genet Mol Res.

[21] Zhao J, Yang C, Guo S, Wu Y (2015). GM130 regulates epithelial-to-mesenchymal transition and invasion of gastric cancer cells via snail. Int J Clin Exp Pathol.

[22] Esquela-Kerscher A, Slack FJ (2006). Oncomirs - microRNAs with a role in cancer. Nat Rev Cancer.

[23] Kent OA, Mendell JT (2006). A small piece in the cancer puzzle: microRNAs as tumor suppressors and oncogenes. Oncogene.

[24] Deng H, Guo Y, Song H, Xiao B, Sun W, Liu Z (2013). MicroRNA-195 and microRNA-378 mediate tumor growth suppression by epigenetical regulation in gastric cancer. Gene.

[25] Browne G, Dragon JA, Hong D, Messier TL, Gordon JA, Farina NH (2016). MicroRNA-378-mediated suppression of Runx1 alleviates the aggressive phenotype of triple-negative MDA-MB-231 human breast cancer cells. Tumour Biol.

[26] Wu QP, Xie YZ, Deng Z, Li XM, Yang W, Jiao CW (2012). Ergosterol peroxide isolated from Ganoderma lucidum abolishes microRNA miR-378-mediated tumor cells on chemoresistance. PLoS One.

[27] Zeng Y, Yi R, Cullen BR (2003). MicroRNAs and small interfering RNAs can inhibit mRNA expression by similar mechanisms. Proc Natl Acad Sci U S A.

[28] Goodwin AM, Kitajewski J, D'Amore PA (2007). Wnt1 and Wnt5a affect endothelial proliferation and capillary length; Wnt2 does not. Growth Factors.

[29] Schwachofer JH, Crooijmans RP, Hoogenhout J, Kal HB, Theeuwes AG (1991). Effectiveness in inhibition of recovery of cell survival by cisplatin and carboplatin: influence of treatment sequence. Int J Radiat Oncol Biol Phys.

[30] Lee HH, Uen YH, Tian YF, Sun CS, Sheu MJ, Kuo HT (2009). Wnt-1 protein as a prognostic biomarker for hepatitis B-related and hepatitis C-related hepatocellular carcinoma after surgery. Cancer Epidemiol Biomark Prev.

